# Epidemiology of vampire bat-transmitted rabies virus in Goiás, central Brazil: re-evaluation based on G-L intergenic region

**DOI:** 10.1186/1756-0500-3-288

**Published:** 2010-11-08

**Authors:** Shinji Hirano, Takuya Itou, Adolorata AB Carvalho, Fumio H Ito, Takeo Sakai

**Affiliations:** 1 Nihon University Veterinary Research Center, 1866 Kameino, Fujisawa 252-0880, Kanagawa, Japan; 2 Faculty of Agriculture and Veterinary Science, Department of Preventive Veterinary Medicine, UNESP, Via de Acesso Prof. Paulo Donato Castellane, Jaboticabal, São Paulo 14884-900, Brazil; 3 Department of Preventive Veterinary Medicine and Animal Health, Faculty of Veterinary Medicine and Zootechny, University of São Paulo, Av. Prof. Dr. Orlando Marques de Paiva, 87, Cidade Universtiátria, São Paulo 05508-000, Brazil

## Abstract

**Background:**

Vampire bat related rabies harms both livestock industry and public health sector in central Brazil. The geographical distributions of vampire bat-transmitted rabies virus variants are delimited by mountain chains. These findings were elucidated by analyzing a high conserved nucleoprotein gene. This study aims to elucidate the detailed epidemiological characters of vampire bat-transmitted rabies virus by phylogenetic methods based on 619-nt sequence including unconserved G-L intergenic region.

**Findings:**

The vampire bat-transmitted rabies virus isolates divided into 8 phylogenetic lineages in the previous nucleoprotein gene analysis were divided into 10 phylogenetic lineages with significant bootstrap values. The distributions of most variants were reconfirmed to be delimited by mountain chains. Furthermore, variants in undulating areas have narrow distributions and are apparently separated by mountain ridges.

**Conclusions:**

This study demonstrates that the 619-nt sequence including G-L intergenic region is more useful for a state-level phylogenetic analysis of rabies virus than the partial nucleoprotein gene, and simultaneously that the distribution of vampire bat-transmitted RABV variants tends to be separated not only by mountain chains but also by mountain ridges, thus suggesting that the diversity of vampire bat-transmitted RABV variants was delimited by geographical undulations.

## Background

Rabies is a zoonosis that kills infected mammals, including humans, and is mainly transmitted by carnivores. In the Americas, chiropterans (insectivorous, frugivorous and hematophagous bat) are another reservoir of this disease. Although dog-transmitted rabies in central Brazil has been reduced by aggressive vaccination programs [1], chiroptera (particularly the common vampire bat, *Desmodus rotundas*)-transmitted rabies remains endemic in this region, and harms both the livestock industry and the public health sector [2,3].

To date, vampire bat-transmitted rabies in livestock has been controlled by reducing the population of vampire bats and by vaccinating livestock [3,4]. However, the depopulation of vampire bats has limitations and the effects are temporary, while vaccination of livestock is only carried out for some animals and is ineffective in decreasing rabies levels in vampire bats.

For the sustainable and effective control of vampire bat rabies, further knowledge of epidemiological features, such as vampire bat ecology and the dynamics of vampire bat-transmitted rabies, is necessary. Molecular epidemiological analysis of vampire bat-transmitted cattle rabies cases using the partial nucleoprotein gene, which is the most conserved gene in the rabies virus (RABV) genome, has suggested that the distribution of variants in Brazil is delimited by mountain chains and clustered in tens of thousands of square kilometers [5]. However, the vampire bats migrate several kilometers from their nests [6]. To elucidate a more detailed genetic clustering and geo-distribution of genetic clades of vampire bat-transmitted RABV, 204 isolates from Goiás, which includes the 185 isolates analyzed previously, were employed and analyzed by a phylogenetic method based on a nucleotide sequence encompassing the G-L intergenic region locating between glycoprotein (G) and polymerase (L) gene loci, which is the most divergent region in the RABV genome and is used for monitoring epidemiological changes in the evolution of RABV [7,8].

## Results

### Phylogenetic analysis

**Figure 1:**
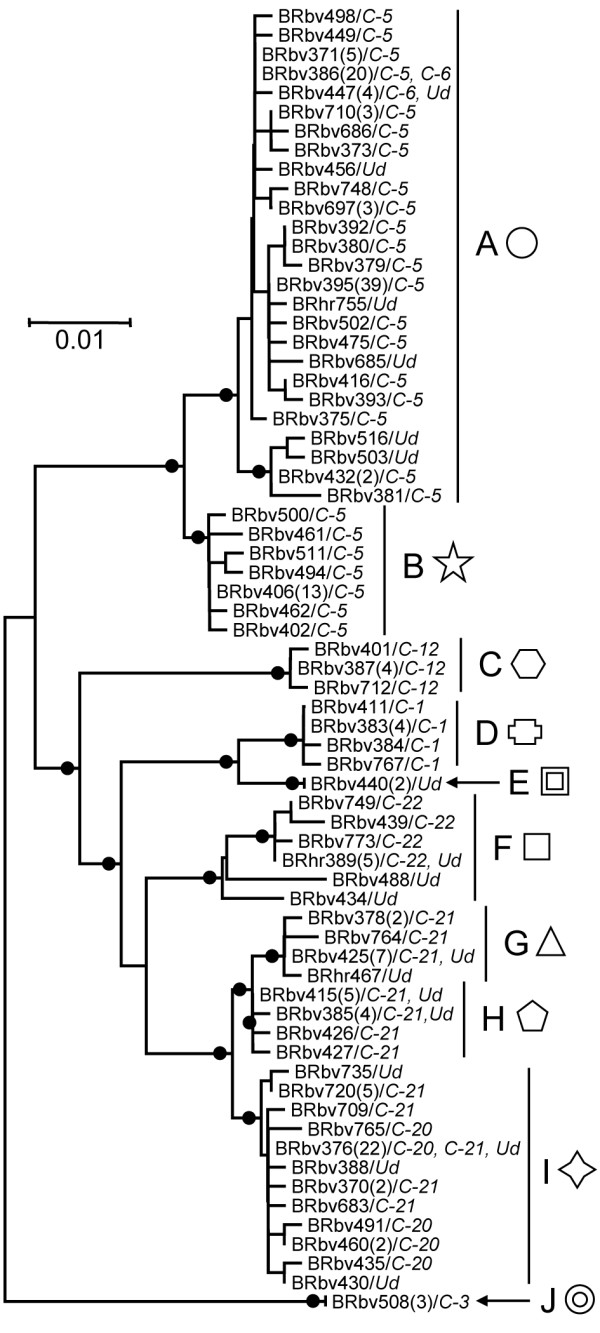
**NJ tree based on 619-nt sequence of the partial G and the G-L intergenic region**. Full circles on internal branches indicate significant bootstrap values (>70%). The number of isolates retaining 100% nucleotide similarity with the representative isolate is shown in parentheses. Lineages of the isolates defined in a previous study [5] are shown in italics after a diagonal (Ud: undefined).

The 204 RABV isolates divided into 8 phylogenetic lineages in the previous nucleoprotein gene analysis were divided into 10 phylogenetic lineages with significant bootstrap values (Figure 1; details shown in Table 1). Isolates of the C-5 and C-6 lineages designated by Kobayashi et al. belonged to the A-lineage, while the B-lineage consisted of some isolates of C-5. The C-, D-, F- and J-lineages included isolates belonging to C-12, C-1, C-22 and C-3, respectively. Isolates of C-20 belonged to the I-lineage, and C-21 was divided into three lineages; G-, H- and I-lineages. Two isolates not belonging to any lineages in previous studies were assigned to the E-lineage.

**Table 1:** Isolates from Goiás

				Grouping		**Accession No**.	
					
Sample	Species	Location	Year	This study	**Previous study**^**c**^	N203-nt	Partial G & GL
BRbv371	Cattle	Caldas Novas	2002	A	C-5	AB307182	AB544082
BRbv372	Cattle	Água Limpa	2002	A	C-5	AB307183	AB544083
BRbv373	Cattle	Caldas Novas	2002	A	C-5	AB307184	AB544084
BRbv375	Cattle	Santa Cruz de Goias	2002	A	C-5	AB307186	AB544085
BRbv379	Cattle	Corumbaíba	2002	A	C-5	AB307190	AB544089
BRbv380	Cattle	Corumbaíba	2002	A	C-5	AB307191	AB544090
BRbv381	Cattle	Cristalina	2002	A	C-5	AB307192	AB544091
BRbv386	Cattle	Ipameri	2002	A	C-5	AB307197	AB544095
BRbv391	Cattle	Caldas Novas	2002	A	C-5	AB307201	AB544099
BRbv392	Cattle	Água Limpa	2002	A	C-5	AB307202	AB544100
BRbv393	Cattle	Itumbiara	2002	A	C-5	AB307203	AB544101
BRbv395	Cattle	Itumbiara	2002	A	C-5	AB307205	AB544102
BRbv396	Cattle	Itumbiara	2002	A	C-5	AB307206	AB544103
BRbv397	Cattle	Buriti Alegre	2002	A	C-5	AB307207	AB544104
BRbv398	Cattle	Itumbiara	2002	A	C-5	AB307208	AB544105
BRbv400	Cattle	Buriti Alegre	2002	A	C-5	AB307210	AB544107
BRbv404	Cattle	Buriti Alegre	2002	A	C-5	AB307214	AB544111
BRbv405	Cattle	Buriti Alegre	2002	A	C-5	AB307215	AB544112
BRbv407	Cattle	Goiandira	2002	A	C-5	AB307217	AB544114
BRbv412	Cattle	Itapaci	2002	A	C-5	AB307222	AB544118
BRbv413	Cattle	Caldas Novas	2002	A	C-5	AB307223	AB544119
BRbv416	Cattle	Morrinhos	2002	A	C-5	AB307226	AB544122
BRbv421	Cattle	Nova Aurora	2002	A	C-5	AB307231	AB544124
BRbv429	Cattle	Corumbaíba	2002	A	Ud^a^	AB307238	AB544129
BRbv432	Cattle	Ipameri	2002	A	C-5	AB307241	AB544132
BRbv438	Cattle	Buriti Alegre	2002	A	C-5	AB307247	AB544137
BRhr441	Horse	Buriti Alegre	2002	A	Ud	AB307251	AB544140
BRbv442	Cattle	Buriti Alegre	2002	A	C-5	AB307252	AB544141
BRbv447	Cattle	Urutaí	2002	A	C-6	AB307255	AB544143
BRbv449	Cattle	Caldas Novas	2002	A	C-5	AB307256	AB544145
BRbv451	Cattle	Ipameri	2002	A	C-5	AB307258	AB544147
BRbv453	Cattle	São Luis de Montes Belos	2002	A	C-5	AB307260	AB544149
BRbv456	Cattle	Orizona	2002	A	Ud	AB307263	AB544150
BRbv457	Cattle	Água Limpa	2002	A	C-5	AB307264	AB544151
BRbv458	Cattle	Buriti Alegre	2002	A	C-5	AB307265	AB544152
BRbv466	Cattle	Itumbiara	2002	A	C-5	AB307273	AB544157
BRbv469	Cattle	Ipameri	2002	A	C-6	AB307275	AB544159
BRbv471	Cattle	Santa Cruz de Goias	2002	A	C-6	AB307277	AB544161
BRbv472	Cattle	Ipameri	2002	A	C-6	AB307278	AB544162
BRbv473	Cattle	Itumbiara	2002	A	C-5	AB307279	AB544163
BRbv475	Cattle	Caldas Novas	2002	A	C-5	AB307281	AB544165
BRbv477	Cattle	Itumbiara	2002	A	C-5	AB307283	AB544166
BRbv478	Cattle	Itumbiara	2002	A	C-5	AB307284	AB544167
BRbv482	Cattle	Panamá	2002	A	C-5	AB307288	AB544170
BRhr483	Horse	Panamá	2002	A	Ud	AB307291	AB544171
BRbv486	Cattle	Itumbiara	2002	A	C-5	AB307293	AB544172
BRbv489	Cattle	Itumbiara	2002	A	Ud	AB307295	AB544174
BRbv493	Cattle	Ipameri	2002	A	C-6	AB307298	AB544177
BRbv495	Cattle	Panamá	2002	A	C-5	AB307300	AB544179
BRbv496	Cattle	Panamá	2002	A	C-5	AB307301	AB544180
BRbv497	Cattle	Itumbiara	2002	A	C-5	AB307302	AB544181
BRbv498	Cattle	Bela Vista de Goiás	2002	A	C-5	AB307303	AB544182
BRbv502	Cattle	Itumbiara	2002	A	C-5	AB307306	AB544186
BRbv503	Cattle	Orizona	2002	A	Ud	AB307307	AB544187
BRbv509	Cattle	Buriti Alegre	2002	A	C-5	AB307311	AB544192
BRbv514	Cattle	Panamá	2002	A	C-5	AB307316	AB544196
BRbv516	Cattle	Orizona	2002	A	Ud	AB307318	AB544198
BRbv521	Cattle	Urutaí	2002	A	Nd^b^	Nd	AB544201
BRbv524	Cattle	Urutaí	2002	A	C-6	AB307323	AB544204
BRbv526	Cattle	Panamá	2002	A	C-5	AB307325	AB544206
BRbv527	Cattle	Campos Belos	2002	A	C-5	AB307326	AB544207
BRbv684	Cattle	Ipameri	2001	A	C-5	AB307412	AB544211
BRbv685	Cattle	Corumbaíba	2001	A	Nd	Nd	AB544212
BRbv686	Cattle	Ipameri	2001	A	C-5	AB307413	AB544213
BRbv687	Cattle	Corumbaíba	2001	A	C-5	AB307414	AB544214
BRbv690	Cattle	Ipameri	2001	A	C-5	AB307417	AB544215
BRbv691	Cattle	Ipameri	2001	A	C-5	AB307418	AB544216
BRbv694	Cattle	Iporá	2001	A	Nd	Nd	AB544219
BRbv697	Cattle	Campo Alegre de Goiás	2001	A	C-5	AB307423	AB544221
BRbv698	Cattle	Campo Alegre de Goiás	2001	A	C-5	AB307424	AB544222
BRbv699	Cattle	Campo Alegre de Goiás	2001	A	C-5	AB307425	AB544223
BRbv700	Cattle	Caldas Novas	2001	A	Nd	Nd	AB544224
BRbv701	Cattle	Caldas Novas	2001	A	C-5	AB307426	AB544225
BRhr704	Horse	Corumbaíba	2001	A	Nd	Nd	AB544227
BRbv705	Cattle	Ipameri	2001	A	C-5	AB307428	AB544228
BRbv707	Cattle	Corumbaíba	2001	A	Nd	Nd	AB544229
BRsp708	Sheep	Ouvidor	2001	A	Nd	Nd	AB544230
BRbv710	Cattle	Morrinhos	2001	A	C-5	AB307431	AB544232
BRbv711	Cattle	Morrinhos	2001	A	C-5	AB307432	AB544233
BRbv717	Cattle	Ipameri	2001	A	C-5	AB307438	AB544235
BRbv718	Cattle	Ipameri	2001	A	C-5	AB307439	AB544236
BRbv741	Cattle	Marzagão	2001	A	C-5	AB307450	AB544244
BRbv742	Cattle	Ipameri	2001	A	C-5	AB307451	AB544245
BRbv745	Cattle	Buriti Alegre	2001	A	C-5	AB307454	AB544248
BRbv747	Cattle	Corumbaíba	2001	A	Ud	AB307456	AB544250
BRbv748	Cattle	Campo Alegre de Goiás	2001	A	C-5	AB307457	AB544251
BRbv752	Cattle	Catalão	2001	A	Ud	AB307461	AB544254
BRhr755	Horse	Cumari	2001	A	Nd	Nd	AB544257
BRbv770	Cattle	Ipameri	2002	A	C-5	AB307476	AB544269
BRbv771	Cattle	Caldas Novas	2002	A	C-5	AB307477	AB544270
BRbv780	Cattle	Nova Crixás	2002	A	C-5	AB307484	AB544274
BRbv785	Cattle	Corumbaíba	2002	A	C-5	AB307486	AB544277
BRbv786	Cattle	Buriti Alegre	2002	A	Ud	AB307487	AB544278
BRbv796	Cattle	Pires do Rio	2002	A	C-6	AB307495	AB544283
BRbv797	Cattle	Cristalina	2002	A	C-6	AB307496	AB544284
BRbv402	Cattle	Divinópolis de Goiás	2002	B	C-5	AB307212	AB544109
BRbv406	Cattle	Posse	2002	B	C-5	AB307216	AB544113
BRbv459	Cattle	Monte Alegre de Goiás	2002	B	C-5	AB307266	AB544153
BRbv461	Cattle	Monte Alegre de Goiás	2002	B	C-5	AB307268	AB544155
BRbv462	Cattle	Monte Alegre de Goiás	2002	B	C-5	AB307269	AB544156
BRbv479	Cattle	Colinas do Sul	2002	B	C-5	AB307285	AB544168
BRbv481	Cattle	Serranópolis	2002	B	C-5	AB307287	AB544169
BRbv490	Cattle	Colinas do Sul	2002	B	C-5	AB307296	AB544175
BRbv494	Cattle	Divinópolis de Goiás	2002	B	C-5	AB307299	AB544178
BRbv500	Cattle	Colinas do Sul	2002	B	C-5	AB307304	AB544184
BRbv505	Cattle	Colinas do Sul	2002	B	C-5	AB307309	AB544188
BRhr506	Horse	Colinas do Sul	2002	B	Nd	Nd	AB544189
BRhr507	Horse	Colinas do Sul	2002	B	Nd	Nd	AB544190
BRbv510	Cattle	Monte Alegre de Goiás	2002	B	C-5	AB307312	AB544193
BRbv511	Cattle	Divinópolis de Goiás	2002	B	C-5	AB307313	AB544194
BRbv518	Cattle	Campos Belos	2002	B	C-5	AB307319	AB544199
BRbv525	Cattle	Panamá	2002	B	C-5	AB307324	AB544205
BRbv753	Cattle	Monte Alegre de Goiás	2001	B	C-5	AB307462	AB544255
BRbv762	Cattle	Divinópolis de Goiás	2001	B	C-5	AB307470	AB544263
BRbv387	Cattle	Doverlândia	2002	C	C-12	AB307198	AB544096
BRbv401	Cattle	Mineiros	2002	C	C-12	AB307211	AB544108
BRbv424	Cattle	Doverlândia	2002	C	C-12	AB307234	AB544125
BRbv692	Cattle	Mineiros	2001	C	C-12	AB307419	AB544217
BRbv712	Cattle	Doverlândia	2001	C	C-12	AB307433	AB544234
BRbv751	Cattle	Mineiros	2001	C	C-12	AB307460	AB544253
BRbv383	Cattle	Nova América	2002	D	C-1	AB307194	AB544092
BRbv384	Cattle	Nova América	2002	D	C-1	AB307195	AB544093
BRbv409	Cattle	Rubiataba	2002	D	C-1	AB307219	AB544115
BRbv411	Cattle	Morrinhos	2002	D	C-1	AB307221	AB544117
BRbv452	Cattle	Rubiataba	2002	D	C-1	AB307259	AB544148
BRbv767	Cattle	Rubiataba	2002	D	C-1	AB307474	AB544267
BRbv793	Cattle	Urutaí	2002	D	C-1	AB307493	AB544282
BRbv440	Cattle	Nova Crixás	2002	E	Ud	AB307249	AB544139
BRbv519	Cattle	Mundo Novo	2002	E	Ud	AB307320	AB544200
BRhr389	Horse	Nova Crixás	2002	F	Nd	Nd	AB544098
BRbv434	Cattle	Itapuranga	2002	F	Ud	AB307243	AB544133
BRbv439	Cattle	Mundo Novo	2002	F	C-22	AB307248	AB544138
BRbv488	Cattle	Carmo do Rio Verde	2002	F	Ud	AB307294	AB544173
BRbv749	Cattle	Nova Crixás	2001	F	C-22	AB307458	AB544252
BRbv757	Cattle	Monte Alegre de Goiás	2001	F	C-22	AB307465	AB544259
BRbv763	Cattle	Aruanã	2002	F	C-22	AB307471	AB544264
BRbv773	Cattle	Aruanã	2002	F	C-22	AB307479	AB544272
BRbv774	Cattle	Mozarlândia	2002	F	C-22	AB307480	AB544273
BRsp787	Sheep	Ipameri	2002	F	Nd	Nd	AB544279
BRbv378	Cattle	Novo Brasil	2002	G	C-21	AB307189	AB544088
BRbv425	Cattle	Novo Brasil	2002	G	C-21	AB307235	AB544126
BRbv445	Cattle	Morrinhos	2002	G	Ud	AB307254	AB544142
BRbv448	Cattle	Buriti de Goiás	2002	G	C-21	AB307256	AB544144
BRhr467	Horse	Buriti de Goiás	2002	G	Nd	Nd	AB544158
BRbv474	Cattle	Moiporá	2002	G	C-21	AB307280	AB544164
BRbv501	Cattle	Anicuns	2002	G	C-21	AB307305	AB544185
BRbv515	Cattle	Córrego do Ouro	2002	G	C-21	AB307317	AB544197
BRbv522	Cattle	Buriti de Goiás	2002	G	Nd	Nd	AB544202
BRbv523	Cattle	Córrego do Ouro	2002	G	C-21	AB307322	AB544203
BRbv764	Cattle	Córrego do Ouro	2002	G	C-21	AB307472	AB544265
BRbv385	Cattle	Ivolândia	2002	H	C-21	AB307196	AB544094
BRbv414	Cattle	Ivolândia	2002	H	C-21	AB307224	AB544120
BRbv415	Cattle	Ivolândia	2002	H	C-21	AB307225	AB544121
BRbv426	Cattle	Amorinópolis	2002	H	C-21	AB307236	AB544127
BRbv427	Cattle	Ivolândia	2002	H	C-21	AB307237	AB544128
BRbv431	Cattle	Palestina de Goiás	2002	H	Ud	AB307240	AB544131
BRbv437	Cattle	Goiandira	2002	H	Ud	AB307246	AB544136
BRbv693	Cattle	Amorinópolis	2001	H	C-21	AB307420	AB544218
BRhr703	Horse	Ivolândia	2001	H	Nd	Nd	AB544226
BRbv736	Cattle	Amorinópolis	2001	H	C-21	AB307446	AB544242
BRbv744	Cattle	Amorinópolis	2001	H	C-21	AB307453	AB544247
BRbv370	Cattle	Caiapônia	2002	I	C-21	AB307181	AB544081
BRbv376	Cattle	Caiapônia	2002	I	C-21	AB307187	AB544086
BRbv377	Cattle	Bom Jardim de Goiás	2002	I	C-21	AB307188	AB544087
BRbv388	Cattle	Piranhas	2002	I	Ud	AB307199	AB544097
BRbv399	Cattle	Rio Verde	2002	I	C-21	AB307209	AB544106
BRbv403	Cattle	Piranhas	2002	I	C-20	AB307213	AB544110
BRbv410	Cattle	Piranhas	2002	I	C-21	AB307220	AB544116
BRbv420	Cattle	Piranhas	2002	I	C-21	AB307230	AB544123
BRbv430	Cattle	Buriti Alegre	2002	I	Ud	AB307239	AB544130
BRbv435	Cattle	Bom Jardim de Goiás	2002	I	C-20	AB307244	AB544134
BRbv436	Cattle	Piranhas	2002	I	Ud	AB307245	AB544135
BRbv450	Cattle	Palestina de Goiás	2002	I	C-21	AB307257	AB544146
BRbv460	Cattle	Caiapônia	2002	I	C-20	AB307267	AB544154
BRbv470	Cattle	Piranhas	2002	I	C-20	AB307276	AB544160
BRbv491	Cattle	Caiapônia	2002	I	C-20	AB307297	AB544176
BRbv499	Cattle	Bom Jardim de Goiás	2002	I	Nd	Nd	AB544183
BRbv513	Cattle	Caiapônia	2002	I	C-20	AB307315	AB544195
BRbv681	Cattle	Piranhas	2001	I	Nd	Nd	AB544208
BRbv682	Cattle	Piranhas	2001	I	Nd	Nd	AB544209
BRbv683	Cattle	Piranhas	2001	I	C-21	AB307411	AB544210
BRbv696	Cattle	Piranhas	2001	I	C-21	AB307422	AB544220
BRbv709	Cattle	Piranhas	2001	I	C-21	AB307430	AB544231
BRbv720	Cattle	Arenópolis	2001	I	C-21	AB307440	AB544237
BRbv721	Cattle	Arenópolis	2001	I	C-21	AB307441	AB544238
BRbv722	Cattle	Arenópolis	2001	I	C-21	AB307442	AB544239
BRbv734	Cattle	Arenópolis	2001	I	C-21	AB307444	AB544240
BRbv735	Cattle	Arenópolis	2001	I	Ud	AB307445	AB544241
BRbv738	Cattle	Piranhas	2001	I	C-20	AB307448	AB544243
BRbv743	Cattle	Marzagão	2001	I	C-20	AB307452	AB544246
BRbv746	Cattle	Caiapônia	2001	I	C-20	AB307455	AB544249
BRbv756	Cattle	Arenópolis	2001	I	C-21	AB307464	AB544258
BRbv758	Cattle	Iporá	2001	I	C-21	AB307466	AB544260
BRbv759	Cattle	Diorama	2001	I	C-21	AB307467	AB544261
BRbv760	Cattle	Palestina de Goiás	2001	I	C-21	AB307468	AB544262
BRbv765	Cattle	Caiapônia	2002	I	C-20	AB307473	AB544266
BRbv769	Cattle	Palestina de Goiás	2002	I	C-21	AB307475	AB544268
BRbv772	Cattle	Arenópolis	2002	I	C-21	AB307478	AB544271
BRhr782	Horse	Palestina de Goiás	2002	I	Nd	Nd	AB544275
BRbv784	Cattle	Piranhas	2002	I	C-20	AB307485	AB544276
BRbv790	Cattle	Pires do Rio	2002	I	C-21	AB307490	AB544280
BRbv508	Cattle	Porangatatu	2002	J	C-3	AB307310	AB544191
BRbv754	Cattle	Alvorada do Norte	2001	J	C-3	AB307463	AB544256
BRbv792	Cattle	Morrinhos	2002	J	C-3	AB307492	AB544281

^a^Undefined; ^b^No data; ^c^Kobayashi et al. (2008)

### Geographical plotting

**Figure 2:**
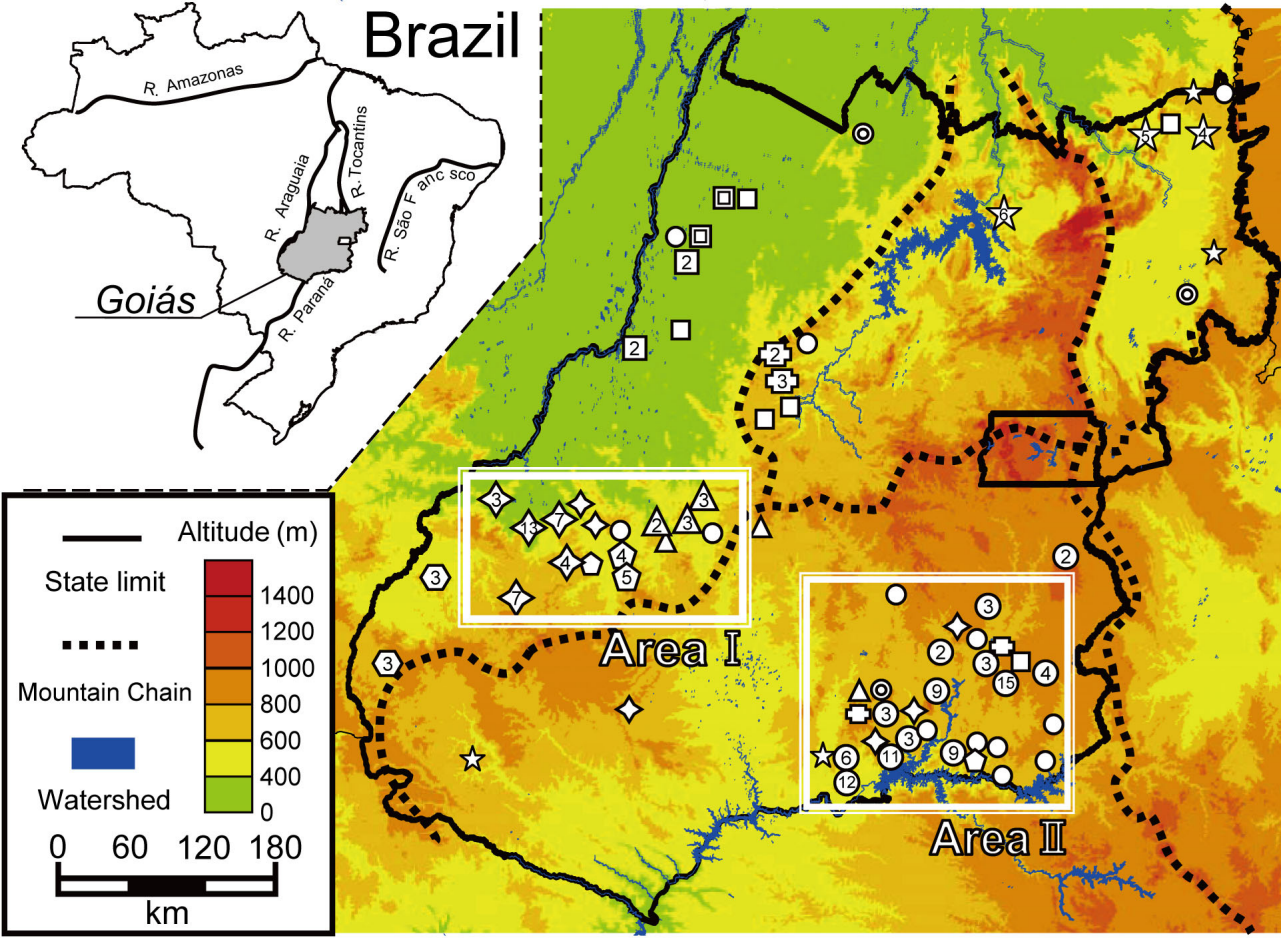
**Distribution of vampire bat-transmitted RABV isolates in Goiás**. Area I: distribution of G-, H- and I-lineages in mountain ridges. Area II: distribution of A-lineage in the basin. Symbols are same as those used in Fig. 1. Numbers inside the symbols indicate the number of isolates obtained in the same region. Small and open symbols indicated that only one isolate was obtained.

In this study, geographical areas in Goiás were divided by mountain chains into the Northwest, North central, Northeast and South regions (Figure 2). Most isolates of the A-lineage were distributed in the South region. The B-lineage was likely to exist in the Northeast and North central regions. The isolates belonging to the C-, E-, F-, G-, H- and I-lineages were distributed in the Northwest region. The G-, H- and I-lineages have narrow distributions and are apparently separated by mountain ridges (Figure 2; Area I), while the A-lineage was distributed in a wide range throughout a Southeastern basin (Figure 2; Area II). The isolates of the D-lineage were plotted on an eastern edge of the North central region. The J-lineage had a wide geographical distribution in Goiás.

## Discussion

Previous studies had elucidated that the distributions of vampire bat and transmitted RABV are delimited by mountain chains [5]. In the present study, it was reconfirmed that mountain chains divide the distribution patterns of each viral lineage. Furthermore, the isolates belonging to C-5 having a wide range in Goiás were divided into the A- and B-lineages, and were found to be distributed in the South and North regions on either side of a mountain chain. This finding supports Kobayashi's hypothesis that distribution of vampire bat-transmitted RABV is affected by mountain chains.

The same variants of vampire bat-transmitted RABV were spread widely in flat low lands (< 800 m), but at higher elevations (800-1600 m), they had a narrower distribution [9]. However, the G-, H- and I-lineages were found to be separated by mountain ridges in low areas (400-800 m) located in a southern undulating area of the Northwestern region (Figure 2; Area I). Furthermore, the A-lineage was located in an eastern basin of the South region (> 800 m; Figure 2; Area II). Considering that the higher lands showed an undulating landscape, the results suggest that the distribution patterns of vampire bat-transmitted RABV variants depend on such undulations. On the other hand, the distribution of common vampire bats in a valley is limited by the ridges that form the valley [10], thus supporting the notion that the distribution of RABV variants is affected by smaller topography than mountain chains.

## Conclusions

The present study analyzed the epidemiology of vampire bat-transmitted RABV using a 619-nt region containing the partial glycoprotein gene and the G-L intergenic region, and indicated that the isolates can be further divided into several phylogenetic lineages with significant bootstrap values when compared to characterization based on the 203-nt partial N gene. Furthermore, the phylogenetic lineages were divided by both mountain chains and mountain ridges. In future studies, it will be important to analyze samples from different time points and to elucidate the dynamics of vampire bat-transmitted rabies in order to establish effective and sustainable control measures for preventing rabies circulation among vampire bats.

## Methods

### Samples

A total of 204 samples obtained from 192 cattle, 10 horses and 2 sheep in Goiás from October 2001 to August 2002 were employed, which had been confirmed as rabies positive through fluorescent antibody test and mouse inoculation test (Table 1). Viral RNA was extracted from the brain as described previously [11]. Lineages of the 164 cattle isolates, C-1, C-3, C-5, C-6, C-12, C-20, C-21 and C-22, were previously characterized based on a 203-nt sequence of the nucleoprotein gene [5], and are shown in Table 1.

### Determination of nucleotide sequences

RT-PCR and direct sequencing with the HmG5-1302 (_4619_TGTTGAAGTTCACCTYCCMGATGT_4642_, positions relative to PV strain genome (Accession No. M13215) and RVLa-1 (_5435_ATRGGGTCATCATAAACCTC_5416_) primer pair were performed as described previously [11]. The target sequence includes the partial glycoprotein gene and G-L intergenic region. Nucleotide sequences were determined using the ATGC program version 4.02 (GENETYX Co., Tokyo, Japan).

### Phylogenetioc analysis

Multiple nucleotide sequence alignments of the partial glycoprotein gene and G-L intergenic region were generated by the ClustalW package in MEGA ver. 4.0 [12]. A phylogenetic tree was constructed by the neighbor joining (NJ) method with bootstrap analysis (1000 psuedoreplicates) under the p-distance model. Phylogenetic clustering supported by a bootstrap value exceeding 70% was regarded as a reliable lineage [13]. Results were validated by the maximum likelihood method using PhyML [14]. In order to reconfirm the shape of the NJ tree, the ML tree was constructed under HKY substitution model justified by MODELTEST packaged in Hyphy program [15].

### Geographical plotting

The 204 RABV isolates were plotted onto a geographical map described using the DIVA-GIS program [16] with GIS data from Instituto Brasileiro de Geografia e Estatística [17] and DIVA-GIS gData [18].

## Competing interests

The authors declare that they have no competing interests.

## Authors' contributions

SH participated in the design of the study, performed the experimental procedures and the data analysis, and wrote the manuscript. TI, AABC, FHI and TS elaborated the study design, management, coordination, and drafting the manuscript. The authors have read and approved the final manuscript.
